# Supplementary material for: Impact of Preprocessing Parameters in Medical Imaging-Based Radiomic Studies: A Systematic Review
Source: Cancers (Basel). 2024 Jul 26;16(15):2668. doi: 10.3390/cancers16152668 (PMC11311340; doi:10.3390/cancers16152668)
Supplement: Supplementary file 1 [file cancers-16-02668-s001.zip › cancers-3093797-supplementary.pdf]

Modality	AUTHOR	YEAR	Number of patients or phantom	Disease	Equipment vendor	Equipment model	Main findings
CBCT	Delgadillo, R. , et al.	2021	30	Prostate cancer	Varian Medical System	Truebeam linear accelerator	Particular reconstruction and preprocessing parameters, adjusted to enhance the consistency of radiomic features in cone-beam computed tomography (CBCT), can inadvertently decrease their reproducibility. Therefore, it is essential to strike a balance between methods that enhance both repeatability and reproducibility of CBCT-based radiomic features. The reconstruction algorithm emerged as the most influential factor affecting the repeatability of these features. Intracranial CBCT (iCBCT) radiomic features tended to be more consistent than spinal CBCT (sCBCT) features. Nevertheless, there is potential for improving the reliability of sCBCT features by applying Collewet normalization, which notably enhances their repeatability.
CT	Linsalata, S. , et al.	2022	20	LARC*	GE Healthcare	LightSpeed RT 16	Preprocessing significantly influences the estimation of textural features in imaging data, with first-order features also being affected to some extent. Preprocessing methods applied before feature extraction can introduce biases in estimating these features.
CT	Au, R. C. , et al.	2021	1210	COPD**	Toshiba<br>Siemens<br>Philips Medical Systems<br>GE Healthcare	Aquilion, Aquilion ONE<br>SOMATOM Definition, SOMATOM Definition AS+, Sensation Cardiac 64<br>BrightSpeed, Discovery CT750 HD, LightSpeed VCT, LightSpeed16<br>Mx8000 IDT 16	The features created through resampling/edgmentation and resampling/thresholding preprocessing methods consistently performed best in classifying Chronic Obstructive Pulmonary Disease (COPD), with an Area Under the Curve (AUC) of at least 0.718. Additionally, these features explained the highest amount of variance in lung function, with an R-squared (R^2) value of at least 0.353.
CT	Nazari, M. , et al.	2021	70	ccRCC***	N.A.	N.A.	SVM: BN_32 (AUCROC 0.79-0.82)better than other BNs. Best performance: LoG _sigma 4.5 and _sigma 5 (AUCROC 0.82-0.84)<br>KNN: BN_32 better (AUCROC 0.86-0.88) than other BNs. Best performance: Wav_HLL (AUCROC 0.86-0.89)<br>GLM: BN_32 better (AUCROC 0.84-0.86) than other BNs. Best performance: Wav_LHL (AUCROC 0.89-0.91)<br>Xgboost: BN_128 better (AUCROC 0.97-0.99) than other BNs. Best performance: BN_128 and Wav_HHH (AUCROC 0.97-0.99)
CT	Kolossváry, M. , et al.	2019	60	Coronary lesions	Philips Medical Systems	Brilliance iCT 256	All volumetric and most radiomic parameters are unchanged using different image reconstruction algorithms.<br>All GLRLM parameters were significantly affected by binning type.<br>BNs significantly affected the values for all GLCM and GLRLM.
CT	Larue, R. , et al.	2017	phantom	N.A.	Philips Medical Systems<br>Siemens	Brilliance 64,Gemini TF 16<br>SOMATOM Definition Flash, Biograph 40, SOMATOM Confidence RT Pro, SOMATOM Sensation Open, SOMATOM Definition Flash, SOMATOM Definition AS+ , Biograph 16	CT-scanner type, slice thickness, and bin width impacted radiomic feature values.<br>Exposure did not show any observable effect on radiomic features.<br>Gray-level discretization can be optimized to enhance prognostic value without jeopardizing feature stability.<br>To resample the images before feature extraction reduces the variability of radiomic features.
CT	Mackin, D. , et al.	2017	8 + phantom (credence cartridge radiomics - CCR)	lung cancer	GE Healthcare	LightSpeed 16 CT	Preprocessing did not affect shape-related features.<br>Resampling combined with filtering improved the Overall Concordance Correlation Coefficient (OCCC) for features derived from First-Order (FO), Gray Level Co-occurrence Matrix (GLCM), Gray Level Run Length Matrix (GLRL), and Neighboring Gray Tone Difference Matrix (NGTDM).<br>A specific bin width setting of 2 and 75 produced the highest feature fraction with OCCC greater than 0.99.<br>Utilizing a resolution of 1 mm per pixel combined with the bin width setting, as mentioned above, reduced the variability introduced by different CT scanners.
CT	Shafiq-Ul-Hassan, M. , et al.	2017	phantom (CCR)	N.A.	GE Healthcare<br>Philips Medical Systems<br>Siemens	Discovery STE, Lightspeed 32 pro<br>Big Bore, Brilliance 64<br>Definition AS, Sensation 64, Sensation 40, Sensation 16	Voxel size resampling is a practical preprocessing step for image datasets acquired with variable voxel sizes, leading to more reproducible CT features.<br>Certain radiomic features showed dependencies on both voxel size and gray-level discretization.<br>Normalizing factors in the definitions of these features significantly reduced or eliminated these dependencies, enhancing their robustness and reproducibility across different voxel sizes and gray-level discretization settings.
CT	Fave, X. , et al.	2016	107	NSCLC	N.A.	N.A.	Smoothing with a specific bin width (BW), either alone or in combination with resampling to an 8-bit depth, most frequently resulted in radiomic features that showed significance in univariate analysis.
CT	Hunter, L. A., et al.	2013	56	NSCLC	GE Healthcare	Discovery ST, LightSpeed RT 16, LightSpeed 16	Average 4D-CT derived image features exhibit superior reproducibility across multiple machines and are thus considered the most suitable candidates for clinical correlation purposes.

Palani, D., et al.	2023	Phantom	N.A.	N.A.	N.A.	The pre-processing filter, CT texture cartridge, and feature category significantly impact the radiomic properties of CCR phantom CT images. GLRLM, GLRLM, and NGTDM feature categories do not statistically alter pre-processing. Different textures (e.g., 30%, 40%, and 50% honeycomb, smooth 3D-printed plaster resin) exhibit regular directional patterns, and most alterations in image pre-processing features are statistically significant in the histogram feature category.<br>Specific pre-processing algorithms, such as Laplacian Filter, Log Filter, Resample, and Bit Depth Rescale Range, significantly influence histogram and GLCM image features.
Stüber, A. T., et al.	2023	530	Hepatic metastases of CRC▪	N.A.	N.A.	60 ML pipeline variations were evaluated on clinical data, and radiomics features from 491 patients. Descriptive analysis of the benchmark results showed a preference for RSF-based pipelines, especially for combining clinical data with radiomics features. The quantitative analysis supported this observation via a linear mixed model approach computed to differentiate the effect of data sets and pipeline configurations on the resulting performance. The RSF pipelines consistently perform similarly to or better than glmnet and xgboost. Further, for the RSF, there was no significantly better-performing pipeline composition regarding preprocessing or hyperparameter optimization.
Demircioğlu, A.	2022	7 datasets	N.A.	N.A.	N.A.	Significant enhancements, up to 0.08 in AUC-ROC, were noted when applying all image preprocessing filters compared to using only original features (up to p = 0.024). Some datasets experienced decreases of -0.04 and -0.10, but these were not statistically significant (p > 0.179). Tuning of image preprocessing filters did not lead to decreases in AUC-ROC. Further improvements of up to 0.1 were observed, although statistically insignificant (p > 0.086), except for one dataset (p = 0.023).
Demircioğlu, A.	2022	7 datasets	N.A.	N.A.	N.A.	Significant enhancements, up to 0.08 in AUC-ROC, were noted when applying all image preprocessing filters compared to using only original features (up to p = 0.024).<br>Some datasets experienced decreases of -0.04 and -0.10, but these were not statistically significant (p > 0.179).<br>Tuning of image preprocessing filters did not lead to decreases in AUC-ROC.<br>Further improvements of up to 0.1 were observed, although statistically insignificant (p > 0.086), except for one dataset (p = 0.023).
Abunahel, B. M., et al.	2022	20 (10 tumor, 10 healthy)	Chronic pancreatitis	Siemens	3 T MAGNETOM Skyra	Thirty-one (28.9%) radiomics features significantly differed between the two groups.<br>These included six shape features, six first-order texture features, and 19 second-order texture features.<br>Kurtosis significantly differed between the two groups after both intensity normalization and discretization.<br>Total energy, energy, mean, root mean squared, 10th percentile, and GLCM correlation significantly differed between the two groups after intensity discretization.
Marfisi, D. , et al.	2022	26	Hypertrophic cardiomyopathy	Siemens	1.5 T MAGNETOM Avanto	Some conventional image preprocessing procedures, such as image resampling/discretization, impacted the sensitivity of myocardial radiomic features from T1 and T2 mapping. This effect was very remarkable only when considering image filtering. Indeed, the sensitivity of radiomic features to different resampling voxel sizes and bin widths was also limited for many textural radiomic features.<br>As for shape and first-order radiomic features, the estimate of most textural radiomic features showed a linear significant correlation with resampling voxel size and bin width. In general, radiomic features from T1 mapping were more sensitive to image preprocessing than radiomic features from T2 mapping, mainly when varying bin width.
Granzier, R. W.	2021	11	N.A.	Philips Medical Systems	1.5 T Ingenia	Images without preprocessing produced the highest number of repeatable features for the T1W sequence and ADC maps, with 15 of 91 (16.5%) and 8 of 91 (8.8%) repeatable features, respectively.<br>Preprocessed images produced between 4 and 14 of 91 and 6 and 7 of 91 repeatable features for T1W and ADC maps.<br>Z-score normalization produced the highest number of repeatable features, 26 of 91 (28.6%), and no preprocessing produced 11 of 91 (12.1%) repeatable features in the T2W sequence.
Hoebel, K. V. , et al.	2021	48	Glioblastoma	Siemens	3 T Tim Trio	Shape features showed higher repeatability than intensity (adjusted P = .001) and texture features (adjusted P = .001) for T2-weighted FLAIR and T1-weighted postcontrast images.<br>Normalization improved the overlap between the region of interest intensity histograms of scan and rescan (adjusted P = .001 for T2-weighted FLAIR and T1-weighted postcontrast images), except in scans where brain extraction fails. As such, normalization significantly improves the repeatability of intensity features from T2-weighted FLAIR scans (adjusted P = .003 [z score normalization] and adjusted P = .002 [histogram matching]).<br>Using a relative intensity binning strategy instead of default absolute intensity binning reduces the correlation between GLCM features after normalization.

Li, Y. , et al.	2021	Phantom + 26 (6 healty, 20 tumors)	Brain tumor	GE Healthcare	Optima MR450w 1.5T, Discovery MR750w 3T	Although intensity normalization methods can make the MRI images standardized and comparable, they cannot remove the scanner effects in the radiomic feature calculation. The ComBat method is essential in removing scanner effects in radiomic features, with the non-biological information removed and the useful biological information preserved. Besides, the intensity normalization methods make the harmonization results more robust when choosing different variants of the ComBat method, namely, standard ComBat, parametric ComBat, and non-parametric ComBat.
McHugh, D. J. , et al.	2021	51	Colorectal Cancer Liver Metastases	Philips Medical Systems	1.5 T Philips Achieva	While most features had non-Gaussian distributions, Box-Cox transformations enabled ICCs and RCs to be calculated appropriately for an average of 97% of features across sequences. Features exhibited a wide range of ICCs, with Shape features having the highest ICCs. 19% of features from non-normalized images exhibited significantly different ICCs in pair-wise comparisons between different MR acquisitions. Using the image normalization tended to increase ICCs for precontrast T1- and T2-weighted images and decrease ICCs for qT1 maps. Finally, RCs and ICCs can provide different insights into feature repeatability.
Carré, A. , et al.	2020	263	Glioma, Gliobalstoma	GE Healthcare	1.5 T Signa EchoSpeed, 3 T Discovery MR750	Nyul's method provided the highest number of robust first-order features based on a threshold value of 0.80 for both ICC and CCC for T1w-gd and T2w-flair sequences, with 16 and 8 features out of 18, respectively. Images without normalization did not generate robust features for the T1w-gd and T2w-flair sequences.
Scalco, E. , et al.	2020	14	Prostate cancer	Philips Medical Systems	1.5 T Philips Achieva	Nearly 60% of the features have shown poor reproducibility (ICC < 0.5) on MRI2, and the method that most affected feature reliability was Norm_ROI (average ICC of 0.45). The other two methods were similar, except for first-order features, where Norm_HM outperformed Norm_Mean (average ICC = 0.33 and 0.76 for Norm_Mean and Norm_HM, respectively). In the inter-observer setting, the number of reproducible features varied in the three structures, higher in the prostate than in the penile bulb and in the obturators. The analysis on Dfeature highlighted that more than 60% of the features were not consistent concerning the normalization method and confirmed the high reproducibility of the features between Norm_Mean and Norm_HM, whereas Norm_ROI was the less reproducible method.
Shiri, I. , et al.	2020	17	Glioblastoma	Siemens	1.5 T SYNGO MR 2004V 4VB11D	The ICC results show high repeatability (ICC ≥ 95%) for image preprocessing, different image registration algorithms, and test-retest analysis. The repeatable features' highest fraction (percent) was observed among registration techniques for the method Full Affine transformation with 12 degrees of freedom using Mutual Information cost function (mean 32.4%) and among image processing methods for the Laplacian of Gaussian (LOG) with Sigma (2.5–4.5 mm) (mean 78.9%). The trends were relatively consistent for N4, N3, or no bias correction. The repeatability performances varied among MR radiomic features for GBM tumors due to test-retest and image registration. The findings have implications for appropriate usage in diagnostic and predictive models.
Simpson, G. , et al.	2020	Phantom + 11	N.A.	N.A.	N.A.	The phantom ROIs quantized to 64 gray level intensities using the histogram equalization method resulted in the greatest number of features with the most minor variability. No precise method resulted in the highest GTV or left kidney repeatability. However, eight texture features extracted from the GTV were repeatable regardless of the ROI processing combination.
Traverso, A. , et al.	2020	81	stage IB–IVA cervical cancer	Siemens	N.A.	The approach using urine-normalized values together with a smaller bin width (0.05) was the most reproducible (428/552, 78% features with ICC >= 0.75); the fixed-bin count approach was the least (215/552, 39% with ICC >= 0.75). Without normalization, using a fixed bin width of 25, 348/552 (63%) of features had an ICC >= 0.75. Overall, 26% (range 25–30%) of the features were volume-dependent (q >= 0.6). None of the volume-independent shape features were reproducible.
Schwier, M. , et al.	2019	15	Prostate Tumor	N.A.	N.A.	The radiomics features, as evaluated on the specific PCa mpMRI dataset, vary significantly in their repeatability. Furthermore, the repeatability of radiomics features evaluated using ICC is highly susceptible to the processing configuration. Even in this small study population, the results already show that the type of image, preprocessing, and region of interest used to evaluate the feature can vastly change the repeatability of certain features. This could explain why feature recommendations among recent studies are inconsistent and why we could not confirm good repeatability for some of the literature-reported features.

MR	Traverso, A. , et al.	2019	56	LARC*	Siemens	N.A.	Inter-observer dependence: features from native ADC maps show consistently high sensitivity, but significant differences are observed for certain feature types (GLSZM and SM) between different datasets and observers. Certain features within a group are less sensitive to differences in delineations.<br>Effect of resampling with interpolation: FO features generally maintain reproducibility with changes in pixel dimensions, except for one feature ("Energy"). GLCM features show moderate reproducibility, while GLSZM features are generally poorly reproducible.<br>Effect of intensity value discretization: FO features, except for Kurtosis and Skewness, are reproducible across various intensity discretization bin widths. However, GLCM and GLSZM features are generally poorly reproducible, except for one GLSZM feature ("GLSZM Gray Level Non-Uniformity").<br>Effect of applying digital image filters: FO features are largely reproducible after additive Gaussian noise. However, the reproducibility of GLCM and GLSZM features varies more with different digital image filters, generally showing poorer reproducibility.
	Um, H. , et al.	2019	161	Glioblastoma	N.A.	N.A.	Radiomic features were more dependent on magnetic field strength than the scannner manufacturer.<br>The shape features were independent of the manufacturer's scanner.<br>Image preprocessing and different binning sizes induce a visible change in covariate shift.<br>All models successfully discriminated between the high-risk and low-risk groups except those constructed with features extracted from MRIs preprocessed with bias field correction and isotropic resampling.
	Brynolfsson, P. , et al.	2017	37(18 glioma, 11 prostate cancer)	Prostate cancer, High-grade Glioma	Siemens	1.5 T Espree	Five imaging and pre-processing parameters are tested: the noise level, resolution, how the ADC map was constructed, the quantization method, and the number of gray levels in the quantized images.<br>Contrast, entropy, and homogeneity change significantly with all varied parameters except the b-values related to ADC map construction. Energy is significantly affected by changes in the number of gray levels, quantization levels, and noise, whereas correlation changes significantly only with resolution and image noise.<br>GLCM size has the most considerable effect on all texture features except for correlation in both data sets and cluster shade and information measure of correlation 1 in the prostate data set.<br>The quantization method significantly affects most features in both data sets.<br>Resolution significantly affects the values for about half of the features.<br>Noise significantly affects all features in the glioma data set, including most features in the prostate data set.<br>The choice of b-values used for constructing the ADC maps in the glioma data set had no significant effect on any feature.
	Abunahel, B., et al.	2022	15 Patients, 15 Control Group	definite chronic pancreatitis	Siemens	MAGNETOM Skyra	Discretization of MR Image Intensities: Using a fixed bin number of 16 resulted in 45 significantly different features. Using a fixed bin number of 128 resulted in 41 significant different features. Using a fixed bin width of 6 resulted in 27 significant different features. Using a fixed bin width of 42 resulted in 29 significant different features.<br>Filtration of MR Images: Using a Laplacian of Gaussian filter with 2mm s resulted in 33 significant different features. Using a Laplacian of Gaussian filter with 5mm s resulted in 5 significantly different features. Using a logarithm filter resulted in 39 significant different features.
	Bleker, J., et al.	2023	930	Prostate Cancer lesions	Philips Healthcare	Ingenia 3T, Achieva 1.5T and Intera 1.5T	The best 2D resampling model (T2W: Bspline and 0.5 mm resolution, DWI: nearest neighbor and 2 mm resolution) significantly outperformed the 2D baseline (AUC: 0.77 vs. 0.64). The best 3D resampling model (T2W: linear and 0.8 mm resolution, DWI: nearest neighbor and 2.5 mm resolution) significantly outperformed the 3D baseline (AUC: 0.79 vs. 0.67).
					Siemens	Skyra 3T, Prisma 3T, Aera 1.5T, Avanto 1.5T and Espree 1.5T	
	Bologna, M., et al.	2023	Phantom	N.A.	Siemens	Magnetom Avanto 1.5T	The use of intensity standardization increased the stability of the first-order statistics features. Shape and size features were always stable for all the analyses. Textural features were susceptible to changes in ST and PS, although some increase in stability could be obtained by voxel size resampling. When images underwent image preprocessing, the number of stable features (ICC > 0.75 and mean absolute CV < 0.3) was 33 for apparent diffusion coefficient (ADC), 52 for T1w, and 73 for T2w.
	Dewi, D., et al.	2023	50	Prostate cancer	Siemens	MAGNETOM Skyra	The pre-processing parameters strongly influenced the reproducibility of radiomics features of T2WI prostate lesions.<br>The setting that yielded the highest number of features (25) with high reproducibility was the relative discretization with a fixed bin number of 64, no signal intensity normalization, and outlier filtering by excluding outliers. Disease characteristics did not significantly impact the reproducibility of radiomics features.

	Foltyn-Dumitru, M., et al.	2023	615	Glioma, IDH	N.A.	N.A.	Internal Test Set: SVM yielded the best performance with macro-average AUCs of 0.84 for naive preprocessing, 0.84 for N4 preprocessing, 0.87 for N4/WS preprocessing, and 0.87 for N4/z-score preprocessing. External Test Sets (UCSF and TCGA): N4/WS and z-score preprocessing approaches outperformed others, with macro-average AUCs ranging from 0.85 to 0.87. This performance replicated that of the internal test set. In contrast, naive preprocessing resulted in macro-average AUCs ranging from 0.19 to 0.45, and N4 alone ranged from 0.26 to 0.52.
	Koçak, B., et al.	2023	50	Brain tumor	N.A.	N.A.	Image preprocessing parameters significantly impacted the segmentation-based reproduc-ibility of radiomic features. The bin width method yielded more reproducible features than the bin count method. In discretization experiments using the bin width on both sequences, according to the ICC cut-off values of 0.75 and 0.90, the rate of reproducible features ranged from 70% to 84% and from 35% to 57%, respectively, with an increasing percentage trend as parameter values de-creased (from 84 to 5 for T2; 100 to 6 for T1ce). The resampling experiments ranged from 53% to 74% and from 10% to 20%, respectively, with an increasing percentage trend from lower to higher parameter values (physical voxel size; from 1x1x1 to 2x2x2 mm^3).
	Marzi, C., et al.	2023	26	Hypertrophic Cardiomyopathy	Siemens	MAGNETOM Avanto,	Using radiomic features extracted from T1 and T2 maps, there is a moderate sensitivity of collinearity analysis and correlation-based dimensionality reduction to some conventional image preprocessing procedures. While, as a whole, this effect is relatively moderate for voxel size resampling and discretization, it is remarkable when considering filtering. Moreover, correlation-based dimensionality reduction is less sensitive to preprocessing when considering radiomic features from T2 compared with T1 maps. This study confirms the need to consider the preprocessing in radiomics toward standardization of methods and when comparing data/results from different clinical studies.
	Salome, P., et al.	2023	338	rHGG,pHGG[◊]	N.A.	N.A.	Variations in the results for the different MR sequences showed that the intensity normalization method performance is sequence-dependent and directly impacts the predictive power of glioma survival models. The normalization approach documentation is highly recommended and necessary to enable the reproducibility of the MRI-based radiomics model.
	Toffoli, T., et al.	2023	28 ccRCC, 20 Oncocytomas	ccRCC***	Philips Medical Systems	Achieva	The univariate analysis incorporating Bonferroni correction did not distinguish any significant features. The pipeline with the highest AUC score (0.83) employed several components, including standard normalization, feature selection based on Random Forest, and a Logistic Regression (LR) classifier with L2 regularization. The top four most frequently selected features were of first order. We identified two groups of correlated features that were uncorrelated with each other. The first group included first-order features (median, 10th percentile, mean, and root mean); the second group consisted of dependence variance (GLDM), large dependence emphasis (GLDM), and long-run emphasis (GLRLM). The classification procedure is based on the best pipeline and uses only two features: 10th percentile (first-order) and dependence variance(GLDM). This approach yielded a mean AUC of 0.90. The K-means algorithm-based unsupervised analysis did not yield significant findings. When the data were divided into two clusters, a 64% correspondence with the oncocytoma/ccRCCclassification was observed, which was less robust than the supervised analysis.
	Ubaldi, L., et al.	2023	461	HGG,LGG[‡]	N.A.	N.A.	The results show that using MRI-reliable features improves the performance in glioma grade classification (AUC = 0.93 ± 0.05) with respect to the use of raw (AUC = 0.88 ± 0.08) and robust features (AUC = 0.83 ± 0.08), defined as those not depending on image normalization and intensity discretization.
	Crandall, J. P. , et al.	2021	14 (17-3)	Cervical Cancer	Siemens	Biograph 40	Good repetability: standard intensity, shape, NGLDM and GLCM
	Ferreira, M. , et al.	2021	158	LACC[†]	Philips Medical Systems Siemens	Gemini TF or BB Biograph mCT	Best results with features discretized with FBN (32 bins) ComBat did not improve the predictive performance of the best models. The performance of the TLR (tumor-to-liver ratio) and native models varied across the scanners used in the test set.
	Xu, H. , et al.	2021	Phantom + 46	Lung disease	Philips Medical Systems	Gemini TF TOF 64	Respiratory motion considerably impacts feature stability in 3D PET imaging, while optimizing preprocessing configuration may improve feature stability and diagnostic performance.
PET/CT	Ger, R. B. , et al.	2019	phantom +224	NSCLC	GE Healthcare	Discovery 710	1) Across all scanners, the average ICC was typically higher with fixed-bin preprocessing than with 64-level preprocessing. 2) Most radiomics feature values had at least good reliability when imaging protocol parameters were within clinically used ranges 3) Interscanner variability was about equal to interpatient variability
	Pfaehler, E. , et al.	2019	phantom: NEMA NU 2-2012 IQ phantom	NSCLC	Siemens	Biograph mCT-40	Images reconstructed with point-spread-function (PSF) resulted in the highest repeatability when compared with OSEM or time-of-flight, for example, 53%, 30%, and 32% of repeatable features, respectively (for unsmoothed data, discretized with FBN, 300 s scan duration). FBW discretization resulted in higher repeatability than FBN discretization, for example, 89% and 35% of the features, respectively (for the EARL-compliant reconstruction and larger high uptake spheres)

* Locally advanced rectal cancer

**Chronic obstructive pulmonary disease

***Clear cell renal cell carcinoma

◊Recurrent and high-grade glioma

‡High- and low-grade glioma

†Locally advanced cervical cancer
